# Perioperative hemodynamic parameters monitored by three noninvasive technologies in children with congenital heart disease: A prospective study

**DOI:** 10.1002/pdi3.2505

**Published:** 2024-09-12

**Authors:** Xiaoyu Xiong, Feng Xu, Wei Qiu, Shaojun Li, Chengjun Liu

**Affiliations:** ^1^ Department of Pediatric Intensive Care Unit Children's Hospital of Chongqing Medical University Chongqing China; ^2^ National Clinical Research Center for Child Health and Disorders Chongqing China; ^3^ Ministry of Education Key Laboratory of Child Development and Disorders Chongqing China; ^4^ China International Science and Technology Cooperation Base of Child Development and Critical Disorders Chongqing China; ^5^ Chongqing Key Laboratory of Pediatrics Chongqing China; ^6^ Department of Emergency Children's Hospital of Chongqing Medical University Chongqing China

**Keywords:** congenital heart disease, echocardiography, electrical cardiometry, hemodynamics, vasoactive inotropic score

## Abstract

This study aims to compare the efficiencies of three noninvasive technologies in monitoring the perioperative hemodynamics of children with congenital heart disease (CHD) including ventricular septal defects with or without atrial septal defects. Three noninvasive technologies included transthoracic echocardiography (TTE), electrical cardiometry (EC), and vasoactive inotropic score (VIS). Parameters included left ventricular ejection fraction (LVEF) and cardiac index (cardiac index monitored by ultrasound, uCI) in TTE, cardiac index (cardiac index monitored by electrical cardiometry, eCI) and systemic vascular resistance index (SVRI) in EC, and VIS. Seventy‐four children were eligible. Three types of adverse events (AEs) related to disease activity and prognosis were observed, including cardio‐pulmonary resuscitation in five cases (5/74, 6.76%), hypoxic‐ischemic brain damage in four cases (4/74, 5.41%) and hemopurification in four cases (4/74, 5.41%). Except for LVEF, eight parameters (VISmax [maximum VIS], VISmea [mean VIS], uCImea [mean uCI], uCImin [minimum uCI], eCImea [mean eCI], eCImin [minimum eCI], SVRImea [mean SVRI], and SVRImin [minimum SVRI]) showed predictive value for any AE (*p* < 0.05). VISmea, uCImea, and eCImea demonstrated the highest accuracy and linear associations (AUROC > 0.9, *p* = 0.00). Linear associations also existed between the three groups of parameters and the duration of mechanical ventilation (MV) and the length of stay (LOS) in the intensive care unit (ICU). The duration of MV and the LOS in the ICU increased as VISmea rose, or uCImea and eCImea fell (*p* < 0.05). LVEF in TTE could not predict any AE (*p* > 0.05) and not fully reflect the cardiovascular function. Therefore, most parameters obtained in TTE, EC, and VIS can reflect the perioperative hemodynamics of children with CHD, with VISmea, uCImea, and eCImea being most accurate.

## Graphical abstract

ROC curves illustrates the predictive value of parameters obtained from three methods in predicting any of the AEs. AEs, adverse events; FPR, false positive rate; TPR, true positive rate; 1 (LVEFmea line overlaps with LVEFmin line); 2 (SVRImea line overlaps with SVRImin line); 3 (SVRImea line, SVRImin line, and LVEFmea line overlap); 4 (SVRImea line overlaps with LVEFmea line); 5 (eCImea line overlaps with eCImin line); 6 (uCImea line overlaps with eCImea line); 7 (uCImea line overlaps with uCImin line); 8 (eCImea line overlaps with uCImin line); 9 (uCImea line, eCImin line, and uCImin line overlap); 10 (uCImea line overlaps with eCImin line); 11 (VISmax line overlaps with VISmea line).

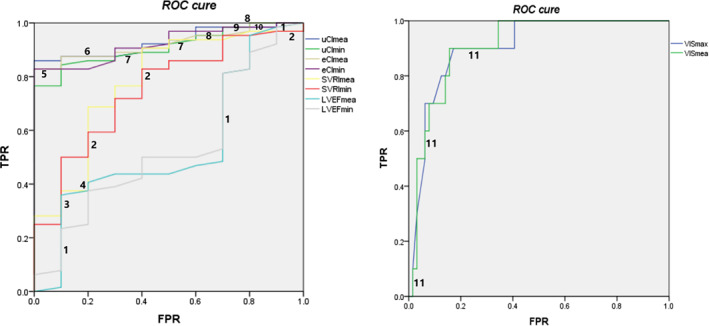

## 1 INTRODUCTION

Congenital heart disease (CHD) occurs in 7–8‰ of children and is a common cause of heart failure.^1^ Ever‐evolving cardiac surgeries under extracorporeal circulation have significantly reduced the morbidity and mortality of heart failure.^2^ However, postoperative hemodynamic instability, such as low cardiac output syndrome, still worsens the prognosis of CHD children.^3^, ^4^, ^5^ Perioperative hemodynamic parameters can be optimized to reduce the risks of heart failure and related deaths and complications, shorten the length of stay (LOS), and recover vital organ functions.^6^, ^7^, ^8^, ^9^, ^10^, ^11^ Therefore, acquiring accurate hemodynamic parameters is critical to improve the disease status and prognosis. Invasive technologies, such as the Swan‐Ganz thermodilution pulmonary artery catheter, are considered a gold standard. However, these technologies may provide deviated data in CHD children presenting cardiac shunt. Complications such as catheter‐vessel mismatch, wound infection, and thrombosis, also limit their wide application.^12^, ^13^ Therefore, noninvasive technologies have attracted increasing interest in the field.

Transthoracic echocardiography (TTE), electrical cardiometry (EC), and vasoactive inotropic score (VIS) have shown value in evaluating children with critical diseases or CHD.^6^, ^12^, ^14^, ^15^ However, their advantages in describing hemodynamic parameters related to disease status and prognosis have never been analyzed. In this study, we compared their performance in monitoring the perioperative hemodynamics of CHD children. We also screened out efficient predictive parameters detected by the three methods, which may be used to improve the perioperative management of CHD children.

## 2 SUBJECTS AND METHODS

### 2.1 Subjects

Infants (aged<1 year) in the perioperative period who had received cardiac surgeries under extracorporeal circulation at the Children's Hospital of Chongqing Medical University from 1 July 2023 to 31 December 2023, were included in the study. CHD mainly included ventricular septal defects with or without atrial septal defects. Operative indications included pulmonary artery systolic pressure ≥40 mmHg and modified Ross score for cardiac function ≥7.

Patients were excluded if any of the following condition is present: complex CHD; pulmonary artery systolic pressure <40 mmHg; Eisenmenger syndrome; modified Ross score for cardiac function <7; unclosed chest during the perioperative period; pneumothorax or subcutaneous emphysema during the perioperative period; use of high‐frequency oscillation mechanical ventilation (MV); severe skin damage or obesity.

## 3 METHODS

### 3.1 Data collection

The clinical profiles of patients during the whole stay in the intensive care unit (ICU) were collected (Table 1). In order to maintain the perfusion and function of the essential organ, each patient received conventional management to keep the invasive radial artery blood pressure above the mean value of the same‐age children reduced by two standard deviations. The VIS was calculated^16^ as VIS = dopamine dose (μg/kg/min) + dobutamine dose (μg/kg/min) + 10 × milrinone dose (μg/kg/min) + 100 × norepinephrine dose (μg/kg/min) + 100 × epinephrine dose (μg/kg/min) + 10,000 × vasopressin dose (Units/kg/min). The VIS was calculated once an hour during the first 24 h after the surgery. Maximum VIS (VISmax) and mean VIS (VISmea) were recorded. The statutory guardians of infants provided written informed consent.

**TABLE 1:** Clinical profiles of 74 CHD children.

Characteristics	Value
Sex	
Male (*n*, %)	39 (52.70%)
Female (*n*, %)	35 (47.30%)
Body weight (kg)	5.40 ± 1.47
Duration of extracorporeal circulation (min)	104 ± 12.55
Duration of aortic occlusion (min)	62 ± 8.35
VISmax	17.10 ± 8.44
VISmea	14.59 ± 6.27
uCImea (L/min.m^2^)	3.64 ± 1.12
uCImin (L/min.m^2^)	2.85 ± 1.04
eCImea (L/min.m^2^)	3.29 ± 0.72
eCImin (L/min.m^2^)	2.53 ± 0.70
SVRImea (cmH_2_O.s/L.m^2^)	1212.32 ± 276.05
SVRImin (cmH_2_O.s/L.m^2^)	1044.18 ± 301.18
LVEFmea (%)	59.22 ± 6.11
LVEFmin (%)	54.29 ± 6.29
AE	
CPR (*n*, %)	5 (6.76%)
Hemopurification (*n*, %)	4 (5.41%)
Hepoxic‐ischemic brain injury (*n*, %)	4 (5.41%)
Duration of MV (h)	34 (20–93)
LOS in the ICU (d)	3 (2–7)

*Note*: Values are Number (%) or arithmetic mean ± 1.96 X standard deviation.

Abbreviations: AE, adverse event; CHD, congenital heart disease; CPR, cardio‐pulmonary resuscitation; eCI, cardiac index detected by electrical cardiometry; ICU, intensive care unit; LOS, length of stay; LVEF, left ventricular ejection fraction; MV, mechanical ventilation; SVRI, systemic vascular resistance index; uCI, cardiac index detected by transthoracic echocardiography; VIS, vasoactive‐inotropic score.

### 3.2 TTE and EC

At 6, 12, 18, 24 h and immediately after surgery under extracorporeal circulation, two ultrasonologists used a portable ultrasound system (M‐Turbo, Sonosite) to record the left ventricular ejection fraction (LVEF) and uCI (uCI = cardiac output/body surface area) from the parasternal long‐axis view and the apical five‐chamber view. For each parameter, its value was measured by one ultrasonologist for three times. The mean of these values was calculated. The two means obtained by two ultrasonologists were further averaged as the final value of this parameter in this round of recording. After five rounds of recording, the minimum LVEF (LVEFmin) and minimum uCI (uCImin), as well as mean LVEF (LVEFmea) and mean uCI (uCImea) were calculated. Meanwhile, another two investigators used EC (AESCULON, OSYPKAMED) to record cardiac index (cardiac index monitored by electrical cardiometry, eCI) and systemic vascular resistance index (SVRI). Their minimums (eCImin, SVRImin) and means (eCImea, SVRImea) were calculated similarly. All investigators were blind to the others' results.

Electrical cardiometry is a bioimpedance technology for continuous (up to 24 h) monitoring of hemodynamic parameters. Four electrodes (black, white, red and green) were pasted respectively on the mid‐forehead, the base of the left neck, the left lateral thorax at the level of xiphoid sternum and the left lateral thigh to monitor the hemodynamic parameters. This technique can monitor more than 10 hemodynamic parameters including cardiac index and peripheral vascular resistance index.

### 3.3 Adverse events (AEs)

As cardio‐pulmonary resuscitation (CPR), hemopurification and hypoxic‐ischemic brain injury often occur in patients with CHD during the perioperative period as results of hemodynamic turbulence, and the occurrence of these events further reflects the tendency of disease aggravation and poor prognosis, representing direct outcome of hemodynamic deterioration. These three clinical events were defined as major adverse events of hemodynamic disturbance in this study.

### 3.4 Statistical analysis

The clinical and perioperative hemodynamic data were analyzed with SPSS 22.0. ROC curves were plotted to evaluate the predictive power of parameters, and correlation analysis was performed to evaluate their associations. *p* < 0.05 was considered statistically significant.

## 4 RESULTS

**FIGURE 1:**
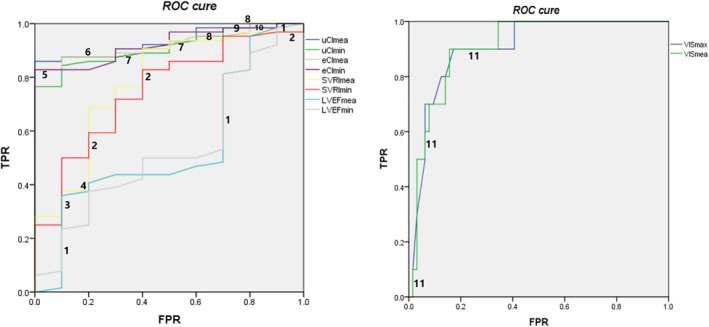
ROC curves illustrating the predictive value of parameters obtained from three methods in predicting any of the AEs. AEs, adverse events; FPR, false positive rate; TPR, true positive rate; 1 (LVEFmea line overlaps with LVEFmin line); 2 (SVRImea line overlaps with SVRImin line); 3 (SVRImea line, SVRImin line, and LVEFmea line overlap); 4 (SVRImea line overlaps with LVEFmea line); 5 (eCImea line overlaps with eCImin line); 6 (uCImea line overlaps with eCImea line); 7 (uCImea line overlaps with uCImin line); 8 (eCImea line overlaps with uCImin line); 9 (uCImea line, eCImin line, and uCImin line overlap); 10 (uCImea line overlaps with eCImin line); 11 (VISmax line overlaps with VISmea line).

**FIGURE 2:**
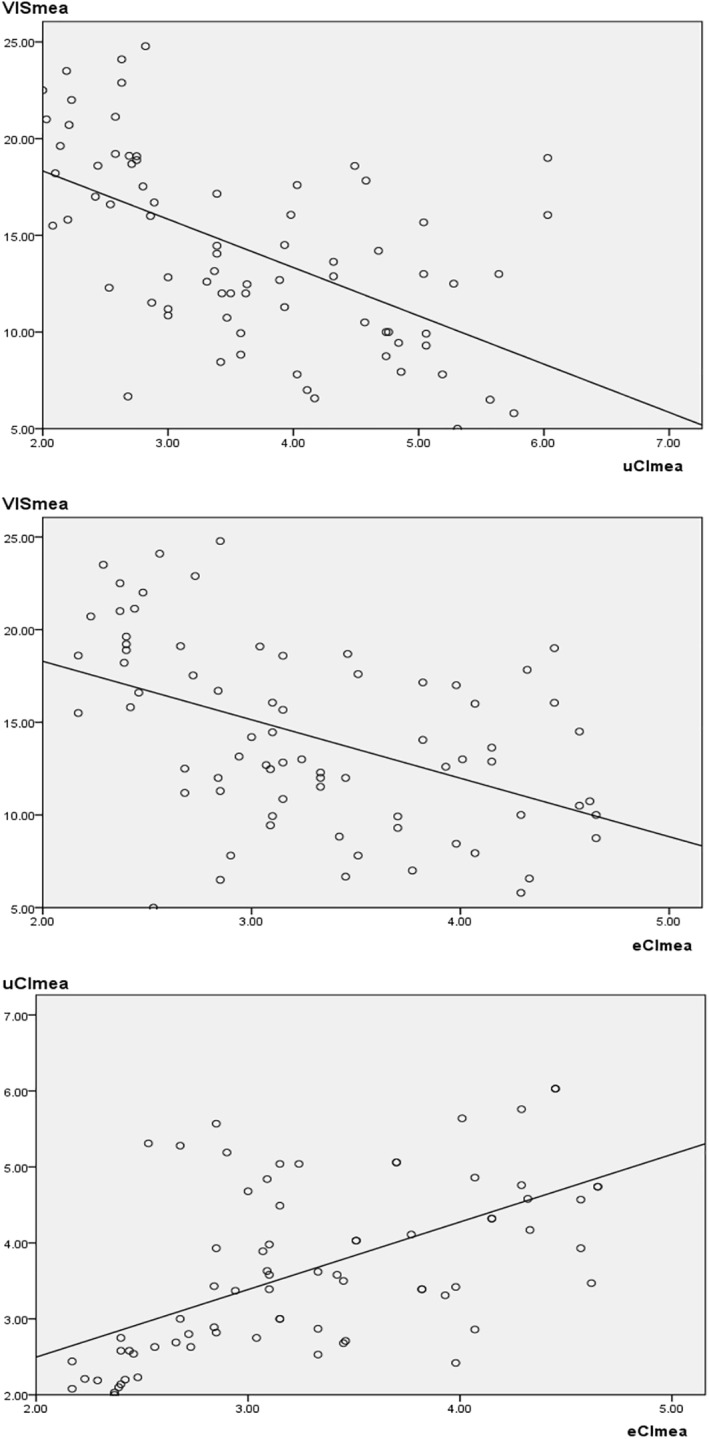
Linear associations between VISmea, uCImea, and eCImea. eCI, cardiac index detected by electrical cardiometry; uCI, cardiac index detected by transthoracic echocardiography; VIS, vasoactive‐inotropic score.

A total of 74 infants were included in this study. Their clinical data and hemodynamic parameters are shown in Table 1. During the perioperative period, three types of AEs were observed, including CPR in five cases (5/74, 6.75%), hypoxic‐ischemic brain injury in four cases (4/74, 5.41%), and hemopurification in four cases (4/74, 5.41%). AEs occasionally occurred in 10 of the 74 cases (10/74, 13.51%). Therefore, we plotted ROC curves to analyze the association between hemodynamic parameters and AEs (Figure 1). We found that VISmax, VISmea, uCImea, uCImin, eCImea, eCImin, SVRImea, and SVRImin were all statistically associated with any AE [VISmax, *p* < 0.01; VISmea, *p* < 0.01; uCImea, *p* < 0.01; uCImin, *p* < 0.01; eCImea, *p* < 0.01; eCImin, *p* < 0.01; SVRImea *p* = 0.01; SVRImin, *p* = 0.01]. Once their values, especially those of VISmea, uCImea, and eCImea, exceeded the J level, the odds of AEs increased significantly, and the prognosis became much worse [VISmax, *p* < 0.01; VISmea, *p* < 0.01; uCImea, *p* < 0.01; uCImin, *p* < 0.01; eCImea, *p* < 0.01; eCImin, *p* < 0.01; SVRImea *p* = 0.01; SVRImin, *p* = 0.01] (Figure 1, Table 2). The parameters with prominent statistical significance were submitted to correlation analysis, which further revealed the linear association between VISmea, uCImea, and eCImea (AUROC [VISmea] = 0.91, *p* < 0.01; AUROC [uCImea] = 0.93, *p* < 0.01; AUROC [eCImea] = 0.92, *p* < 0.01; correlation coefficient [VISmea‐uCImea, *p* < 0.01; VISmea‐eCImea, *p* < 0.01; uCImea‐eCImea, *p* < 0.01]) (Figure 2, Table 3). Therefore, it can be considered that the three parameters achieved consistent results in evaluating the perioperative hemodynamics and prognosis. However, LVEF, as a conventional indicator of contractile function, showed no statistical significance in predicting any of the AEs (AUROC [LVEFmea] = 0.54, *p* = 0.70; AUROC [LVEFmin] = 0.53, *p* = 0.73) (Figure 1, Table 2). It could not fully reflect the disease and cardiovascular functional statuses of CHD children during the perioperative period.

**TABLE 2:** Areas under curves and J values of parameters detected by three methods in predicting any of the AEs.

	AUROC	Standard error	*p*	95% CI		J	Sensitivity	Specificity
				Lower limit	Upper limit			
VISmax	0.91	0.04	<0.01	0.82	0.99	19.50	90%	82.8%
VISmea	0.91	0.04	<0.01	0.83	0.99	18.02	90%	84.4%
uCImea	0.93	0.03	<0.01	0.87	0.99	2.70	85.9%	100%
uCImin	0.91	0.04	<0.01	0.84	0.98	2.07	76.6%	100%
eCImea	0.92	0.03	<0.01	0.86	0.98	2.79	82.8%	100%
eCImin	0.92	0.03	<0.01	0.86	0.99	1.99	82.8%	100%
SVRImea	0.78	0.08	0.01	0.62	0.94	1008.50	90.6%	40%
SVRImin	0.75	0.08	0.01	0.60	0.90	897.50	82.8%	40%
LVEFmea	0.54	0.10	0.70	0.34	0.73	61.06	35.9%	10%
LVEFmin	0.53	0.10	0.73	0.34	0.72	55.75	37.5%	20%

Abbreviations: AE, adverse event; AUROC, areas under the ROC curve; eCI, cardiac index detected by electrical cardiometry; LVEF, left ventricular ejection fraction; SVRI, systemic vascular resistance index; uCI, cardiac index detected by transthoracic echocardiography; VIS, vasoactive‐inotropic score.

**TABLE 3:** Correlation coefficients between VISmea, uCImea, and eCImea.

Dependent variables (*Y*)–independent variables (*X*)	*b*	*R* ^2^	*F*	*p*	Constant
VISmea‐uCImea	−2.50	0.32	34.54	<0.01	23.33
VISmea‐eCImea	−3.16	0.22	20.11	<0.01	24.60
uCImea‐eCImea	0.89	0.33	36.12	<0.01	0.72

Abbreviations: eCI, cardiac index detected by electrical cardiometry; uCI, cardiac index detected by transthoracic echocardiography; VIS, vasoactive‐inotropic score.

**FIGURE 3:**
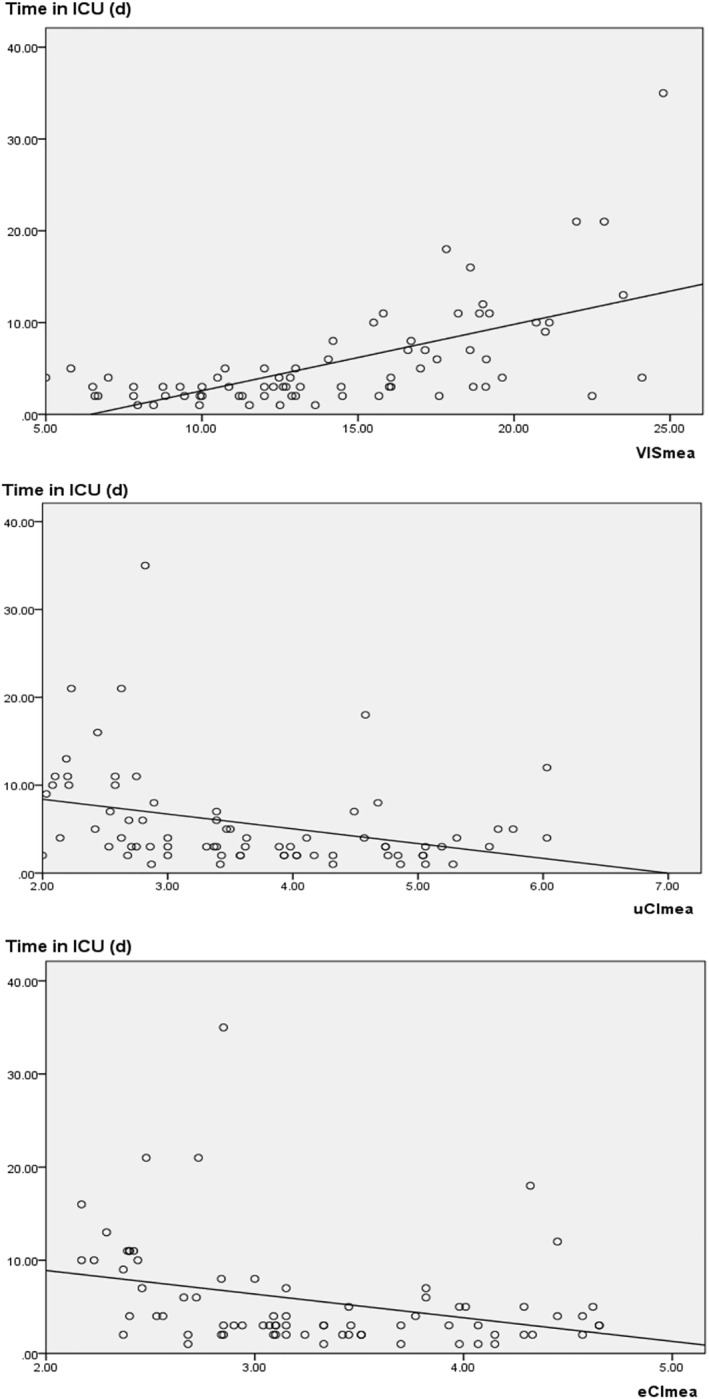
Linear relationships of LOS in the ICU with VISmea, uCImea, and eCImea. eCI, cardiac index detected by electrical cardiometry; ICU, intensive care unit; LOS, length of stay; uCI, cardiac index detected by transthoracic echocardiography; VIS, vasoactive‐inotropic score.

**FIGURE 4:**
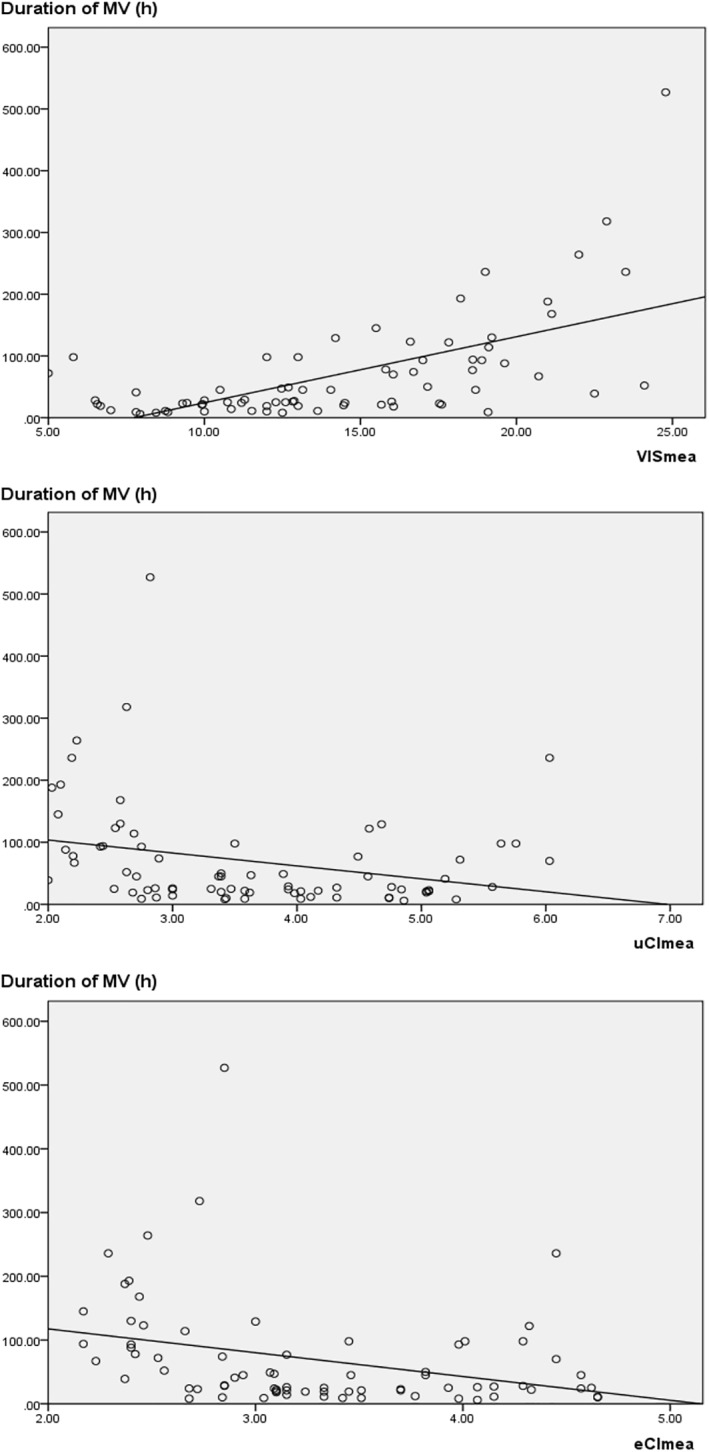
Linear relationships of the duration of MV with VISmea, uCImea, and eCImea. eCI, cardiac index detected by electrical cardiometry; MV, mechanical ventilation; uCI, cardiac index detected by transthoracic echocardiography; VIS, vasoactive‐inotropic score.

We also found that the duration of MV and LOS in the ICU prolongated as VISmea increased or uCImea and eCImea decreased, with a linear association between them (correlation coefficient [Duration of MV‐VISmea, *p* < 0.01; Duration of MV‐uCImea, *p* = 0.02; Duration of MV‐eCImea, *p* = 0.01; LOS in the ICU‐VISmea, *p* < 0.01; LOS in the ICU‐uCImea, *p* < 0.01; LOS in the ICU‐eCImea, *p* = 0.01]) (Figures 3 and 4, Table 4). These findings also supported the value of hemodynamic parameters obtained by three noninvasive tools in predicting disease status and prognosis.

**TABLE 4:** Correlation coefficients between LOS in the ICU, duration of MV and VISmea, uCImea, and eCImea.

Dependent variables (*Y*)–independent variables (*X*)	*b*	*R* ^2^	*F*	*p*	Constant
LOS in the ICU‐VISmea	0.72	0.34	45.64	<0.01	−4.67
LOS in the ICU‐uCImea	−1.68	0.11	8.79	<0.01	11.76
LOS in the ICU‐eCImea	−2.54	0.11	8.41	0.01	13.97
Duration of MV‐VISmea	10.70	0.38	43.55	<0.01	−82.82
Duration of MV‐uCImea	−20.83	0.07	5.77	0.02	145.25
Duration of MV‐eCImea	−37.34	0.10	8.07	0.01	192.15

Abbreviations: eCI, cardiac index detected by electrical cardiometry; ICU, intensive care unit; LOS, length of stay; MV, mechanical ventilation; uCI, cardiac index detected by transthoracic echocardiography; VIS, vasoactive‐inotropic score.

## 5 DISCUSSION

Congenital heart disease is a common disease that can cause severe or critical symptoms. Disrupted hemodynamics may occur during the perioperative period of extracorporeal circulation, which further worsens the condition and prognosis of patients. In these cases, vasoactive agents are usually indicated. It has been reported that VIS, as a weighted sum of all medicated vasoactive agents, can be used to evaluate the disease status and prognosis after cardiac surgery, thus making it a parameter for evaluating cardiac dysfunction or hemodynamic disorders.^1^, ^15^, ^16^, ^17^, ^18^, ^19^, ^20^, ^21^ In the present study, a higher VIS within perioperative 24 h predicted a higher risk of AEs, longer LOS in the ICU and duration of MV (Figures 1, 3 and 4; Tables 2 and 4). In addition, some critical values, such as VISmax (19.50) and VISmea (18.02), exhibited high sensitivity and specificity (Table 2). Similar to the cardiac indexes obtained from the other two techniques, VIS demonstrated a strong ability to predict the risk of clinical AEs (AUROC [VISmax] = 0.91, *p* < 0.01; AUROC [VISmea] = 0.91, *p* < 0.01). Besides, VIS was superior to SVRI (detected by EC) and LVEF (detected by TTE) (Figure 1, Table 2). Therefore, our results also proved that VIS could accurately reflect the severity of the hemodynamic disorder. However, VIS is routinely used to evaluate the general hemodynamic situation without giving the value of each parameter. We introduced the other two noninvasive techniques and compared their results with VIS.

As a routine tool for monitoring cardiac function in pediatric patients, echocardiography is commonly used to diagnose heart failure.^6^, ^22^ In the present study, however, we found no evident association between LVEF and disease severity or cardiovascular function in CHD children (Figure 1, Table 2). An explanation may be that after ventricular septal defect repair, the value of LVEF may be errored by the presence of left ventricle segmental systolic function disorder or ventricular shunt, which cannot reflect the cardiac pump function. However, the uCI detected in the present study escaped this effect, because the cardiac index of the left ventricular outflow tract was directly measured based on the blood flowing into the peripheral organs. We found that a lower uCI parameter indicated a higher risk of AE and a longer treatment. Particularly, uCImea <2.70/min·m^2^ or uCImin <2.07 L/min·m^2^ could achieve a specificity of 100%. Among all the parameters, uCImea manifested the highest accuracy in predicting AEs (AUROC 0.93, *p* < 0.01) (Figures 1, 3 and 4; Tables 2 and 4). These results further verified the superiority of uCI to other indexes acquired by noninvasive techniques in evaluating the hemodynamics of CHD children. Therefore, some scholars have advocated that uCI can be used as a standard to evaluate the target organ perfusion and oxygen supply in hemodynamic disorders and is associated with early status and prognosis of the disease.^23^ However, the application of TTE is sometimes limited by various factors (such as complicated manipulation), making it difficult to perform continuous monitoring with TTE. Therefore, we took EC parameters as reference.

Electrical cardiometry is a bioimpedance technology for continuous (up to 24 h) monitoring of hemodynamic parameters. It has been suggested that the cardiac output detected by EC is not as accurate as that detected by invasive technology or TTE, even lower than the actual cardiac output.^6^, ^12^, ^13^, ^24^ However, to overcome the disadvantage of accurate parameters, EC has been increasingly recommended to continuously monitor hemodynamic parameters and their trends during the intervention.^6^, ^12^, ^14^, ^25^, ^26^ In the present study, cardiac indexes detected by EC and those detected by TTE showed linear relationships with VIS. All three parameters (eCI, uCI, and VIS) obtained by three methods showed similar accuracy in predicting any of the AEs, LOS of ICU, and duration of MV (Figures 1, 2, 3, 4; Tables 2, 3, 4). Therefore, it can be considered that three parameters (eCI, uCI, and VIS) underwent similar trends and could reflect the disease status and prognosis. However, we found that eCI was lower than uCI detected at the same time (Table 1; eCImea and uCImea, *p* = 0.02; eCImin and uCImin, *p* = 0.03), which was consistent with the previous result about cardiac output,^6^, ^12^, ^13^, ^24^ and possibly related with factors like EC methodology or chest wall thickness. Besides, SVRI detected by EC could also reflect the severity and prognosis of the disease, but its accuracy was lower than that of other parameters (AUROC [SVRImea] = 0.78, *p* = 0.01; AUROC [SVRImin] = 0.75, *p* = 0.01) (Figure 1; Table 2). EC possibly reflects the hemodynamics in the thorax and cannot fully reflect the hemodynamics in all the organs, thus affecting the accuracy of SVRI in predicting the disease status and prognosis. Data on EC and SVRI are scarce, and more in‐depth studies are expected. In summary, continuous parameters obtained by EC can display the changes in hemodynamics, showing significant association with those detected by the other two methods. In addition, EC is safe, reproducible, and more comprehensive, making it a valuable complement to TTE.^26^

The present study is novel in that three noninvasive methods were simultaneously used to monitor the hemodynamics in CHD children. This monitoring pattern integrates the comprehensiveness of VIS, the accuracy of TTE, and the continuousness of EC. VISmea, uCImea, and eCImea are all linearly intercorrelated, and three parameters are more accurate in predicting any of the AEs (Figures 1 and 2, Tables 2 and 3). Therefore, their combination is more accurate in continuously monitoring the hemodynamics of CHD children. However, this study is limited by its single‐center design and small sample size. Future larger‐sample, multi‐center studies should yield more reliable results.

## 6 CONCLUSIONS

Parameters detected by TTE, EC, and VIS (especially VISmea, uCImea, and eCImea, but not LVEF) can accurately manifest the perioperative hemodynamics and predict the outcomes of CHD children. Their combination has the potential to better describe the hemodynamics of this population in the clinical setting.

## AUTHOR CONTRIBUTIONS

Xiaoyu Xiong contributed to conception, design, analysis and drafted manuscript; Feng Xu contributed to interpretation and critically revised manuscript; Chengjun Liu contributed to analysis and critically revised manuscript; Shaojun Li contributed to interpretation and critically revised manuscript; Wei Qiu contributed to acquisition and critically revised manuscript.

## CONFLICT OF INTEREST STATEMENT

The authors declare that they have no competing interest.

## ETHICS STATEMENT

The authors declare that the work is written with due consideration of ethical standards. The study was approved by Children's Hospital of Chongqing Medical University (NO. 2020‐112). All methods were performed in accordance with the ethical standards as laid down in the Declaration of Helsinki and its later amendments or comparable ethical standards.

## CONSENT TO PARTICIPATE

The statutory guardians of infants provided written informed consent.

## Data Availability

The datasets used and/or analyzed during the current study are available from the corresponding author on reasonable request.

## References

[1] Garcia RU , Walters HL , Delius RE , Aggarwal S . Vasoactive inotropic score (VIS) as biomarker of short‐term outcomes in adolescents after cardiothoracic surgery. Pediatr Cardiol. 2016;37(2):271‐277. 26424215 10.1007/s00246-015-1273-7

[2] Pieri M , Belletti A , Monaco F , et al. Outcome of cardiac surgery in patients with low preoperative ejection fraction. BMC Anesthesiol. 2016;16(1):97. 27760527 10.1186/s12871-016-0271-5 PMC5069974

[3] Balderas‐Muñoz K , Rodríguez‐Zanella H , Fritche‐Salazar JF , et al. Improving risk assessment for post‐surgical low cardiac output syndrome in patients without severely reduced ejection fraction undergoing open aortic valve replacement. The role of global longitudinal strain and right ventricular free wall strain. Int J Cardiovasc Imag. 2017;33(10):1483‐1489. 10.1007/s10554-017-1139-6 28488096

[4] Pérez VJL , Jiménez Rivera JJ , Alcalá Llorente MA , et al. Low cardiac output syndrome in the postoperative period of cardiac surgery. Profile, differences in clinical course and prognosis. The ESBAGA study. Med Intensiva. 2018;42:159‐167. 28736085 10.1016/j.medin.2017.05.009

[5] Butts RJ , Scheurer MA , Atz AM , et al. Comparison of maximum vasoactive inotropic score and low cardiac output syndrome as markers of early postoperative outcomes after neonatal cardiac surgery. Pediatr Cardiol. 2012;33(4):633‐638. 22349666 10.1007/s00246-012-0193-z PMC3989285

[6] Sanders M , Servaas S , Slagt C . Accuracy and precision of non‐invasive cardiac output monitoring by electrical cardiometry: a systematic review and meta‐analysis. J Clin Monit Comput. 2020;34(3):433‐460. 31175501 10.1007/s10877-019-00330-y PMC7205855

[7] Cecconi M , Corredor C , Arulkumaran N , et al. Clinical review: goal‐directed therapy‐what is the evidence in surgical patients? The effect on different risk groups. Crit Care. 2013;17(2):209. 23672779 10.1186/cc11823 PMC3679445

[8] Sun Y , Chai F , Pan C , Romeiser JL , Gan TJ . Effect of perioperative goal‐directed hemodynamic therapy on postoperative recovery following major abdominal surgery‐a systematic review and meta‐analysis of randomized controlled trials. Crit Care. 2017;21(1):141. 28602158 10.1186/s13054-017-1728-8 PMC5467058

[9] Grocott MP , Dushianthan A , Hamilton MA , Mythen M , Harrison D , Rowan K . Perioperative increase in global blood flow to explicit defined goals and outcomes after surgery: a Cochrane Systematic Review. Br J Anaesth. 2013;111(4):535‐548. 23661403 10.1093/bja/aet155

[10] Hamilton MA , Cecconi M , Rhodes A . A systematic review and meta‐analysis on the use of preemptive hemodynamic intervention to improve postoperative outcomes in moderate and high‐risk surgical patients. Anesth Analg. 2011;112(6):1392‐1402. 20966436 10.1213/ANE.0b013e3181eeaae5

[11] Pearse RM , Harrison DA , MacDonald N , et al. Effect of a perioperative, cardiac output‐guided hemodynamic therapy algorithm on outcomes following major gastrointestinal surgery: a randomized clinical trial and systematic review. JAMA. 2014;311(21):2181‐2190. 24842135 10.1001/jama.2014.5305

[12] Schubert S , Schmitz T , Weiss M , et al. Continuous, non‐invasive techniques to determine cardiac output in children after cardiac surgery: evaluation of transesophageal Doppler and electric velocimetry. J Clin Monit Comput. 2008;2(4):299‐307. 10.1007/s10877-008-9133-0 18665449

[13] Tirotta CF , Lagueruela RG , Madril D , et al. Non‐invasive cardiac output monitor validation study in pediatric cardiac surgery patients. J Clin Anesth. 2017;38:129‐132. 28372651 10.1016/j.jclinane.2017.02.001

[14] Noori S , Drabu B , Soleymani S , Seri I . Continuous non‐invasive cardiac output measurements in the neonate by electrical velocimetry: a comparison with echocardiography. Arch Dis Child Fetal Neonatal Ed. 2012;97(5):F340‐F343. 22933092 10.1136/fetalneonatal-2011-301090

[15] Musick MA , Loftis LL , Kennedy CE . Comparing vasoactive‐inotropic score reporting strategies in the PICU relative to mortality risk. Pediatr Crit Care Med. 2018;19(12):1130‐1136. 30520839 10.1097/PCC.0000000000001738

[16] Gaies MG , Gurney JG , Yen AH , et al. Vasoactive‐inotropic score as a predictor of morbidity and mortality in infants after cardiopulmonary bypass. Pediatr Crit Care Med. 2010;11(2):234‐238. 19794327 10.1097/PCC.0b013e3181b806fc

[17] Han J , Pinsino A , Sanchez J , et al. Prognostic value of vasoactive‐inotropic score following continuous flow left ventricular assist device implantation. J Heart Lung Transplant. 2019;38(9):930‐938. 31201088 10.1016/j.healun.2019.05.007 PMC9891263

[18] Kawai Y , Cornell TT , Cooley EG , et al. Therapeutic plasma exchange may improve hemodynamics and organ failure among children with sepsis‐induced multiple organ dysfunction syndrome receiving extracorporeal life support. Pediatr Crit Care Med. 2015;16(4):366‐374. 25599148 10.1097/PCC.0000000000000351 PMC4424057

[19] Conlon TW , Falkensammer CB , Hammond RS , Nadkarni VM , Berg RA , Topjian AA . Association of left ventricular systolic function and vasopressor support with survival following pediatric out‐of‐hospital cardiac arrest. Pediatr Crit Care Med. 2015;16(2):146‐154. 25560427 10.1097/PCC.0000000000000305 PMC4315701

[20] Aziz KB , Lavilla OC , Wynn JL , Lure AC , Gipson D , de la Cruz D . Maximum vasoactive‐inotropic score and mortality in extremely premature, extremely low birth weight infants. J Perinatol. 2021;41(9):2337‐2344. 33712712 10.1038/s41372-021-01030-9 PMC8435049

[21] Dilli D , Akduman H , Orun UA , et al. Predictive value of vasoactive‐inotropic score for mortality in newborns undergoing cardiac surgery. Indian Pediatr. 2019;56(9):735‐740. 31638004

[22] Lauritsen J , Gustafsson F , Abdulla J . Characteristics and long‐term prognosis of patients with heart failure and mid‐range ejection fraction compared with reduced and preserved ejection fraction: a systematic review and meta‐analysis. ESC Heart Fail. 2018;5(4):685‐694. 29660263 10.1002/ehf2.12283 PMC6073025

[23] Abdalaziz FA , Algebaly HAF , Ismail RI , El‐Sherbini SA , Behairy A . The use of bedside echocardiography for measuring cardiac index and systemic vascular resistance in pediatric patients with septic shock. Rev Bras Ter Intensiva. 2018;30(4):460‐470. 30672970 10.5935/0103-507X.20180067 PMC6334480

[24] Cox PBW , Ouden AMD , Theunissen M , et al. Accuracy, precision, and trending ability of electrical cardiometry cardiac index versus continuous pulmonary artery thermodilution method: a prospective, observational study. BioMed Res Int. 2017;2017:2635151. 29130036 10.1155/2017/2635151 PMC5654291

[25] Tomaske M , Knirsch W , Kretschmar O , et al. Evaluation of the Aesculon cardiac output monitor by subxiphoidal Doppler flow measurement in children with congenital heart defects. Eur J Anaesthesiol. 2009;26:412‐415. 19276980 10.1097/EJA.0b013e3283240438

[26] Yoshitake S , Miyamoto T , Tanaka Y , Naito Y . Non‐invasive measurement of cardiac output using AESCULON^®^ mini after Fontan operation. Pediatr Int. 2017;59(2):141‐144. 27378014 10.1111/ped.13084

